# Distinctive Immunological Signatures Define Foot‐and‐Mouth Disease Virus Persistence in Vaccinated Cattle

**DOI:** 10.1155/tbed/4010309

**Published:** 2025-12-28

**Authors:** Zhihui Zhang, Zhidong Teng, Shuang Wang, Suyu Mu, Sumin Wei, Hu Dong, Shuanghui Yin, Yun Zhang, Yaozhong Ding, Yijing Li, Shiqi Sun, Huichen Guo

**Affiliations:** ^1^ State Key Laboratory for Animal Disease Control and Prevention, College of Veterinary Medicine, Lanzhou University, Lanzhou Veterinary Research Institute, Chinese Academy of Agricultural Sciences, Lanzhou, China, caas.cn; ^2^ College of Veterinary Medicine, Northeast Agricultural University, Harbin, China, neau.edu.cn; ^3^ Yunnan Tropical and Subtropical Animal Virus Diseases Laboratory, Yunnan Animal Science and Veterinary Institute, Kunming, China; ^4^ College of Veterinary Medicine, Gansu Agricultural University, Lanzhou, China, gsau.edu.cn; ^5^ School of Life Sciences, Ningxia University, Yinchuan, China, nxu.edu.cn

## Abstract

Persistent infection with foot‐and‐mouth disease virus (FMDV) develops in over 50% of infected ruminants, presenting major obstacles to disease control and eradication. To clarify host immune correlates of FMDV persistence, we characterized systemic T‐ and B‐cell responses, as well as mucosal responses in 15 vaccinated cattle following experimental FMDV challenge. The prevalence of FMDV persistence was 53.3%. While peripheral CD4^+^, CD8^+^, and *γδ* T‐cell populations and their respective naïve/memory/effector subpopulations showed comparable frequencies between carriers and noncarriers, carriers exhibited significantly lower frequencies of IFN‐*γ*‐producing CD4^+^ and CD8^+^ T lymphocytes during early infection, indicating compromised cell‐mediated immune responses essential for viral clearance. During persistent infection, carriers displayed a distinctive immunological profile characterized by significantly reduced peripheral B‐cell frequencies and increased secretory IgA (sIgA) levels in oropharyngeal fluid (OPF), with comparable systemic antigen‐specific and neutralizing antibody titers across groups throughout the study period. Notably, the combination of peripheral B cell frequencies with OPF sIgA levels demonstrated superior diagnostic specificity for vaccinated carrier identification compared to either indicator alone. Our findings highlight key immune features of FMDV persistence and propose a dual‐biomarker approach for detecting asymptomatic carriers.

## 1. Introduction

Foot‐and‐mouth disease virus (FMDV), the causative agent of foot‐and‐mouth disease (FMD), poses significant challenges to animal health management due to its exceptional contagiousness and broad cloven‐hoofed host range [1, 2]. The disease imposes substantial economic losses on livestock industries through reduced productivity and stringent trade restrictions in affected regions. FMDV’s rapid replication and high mutation rate generate diverse viral populations exhibiting limited intra‐serotype and intra‐strain cross‐protection [2–4], complicating vaccine development and disease control strategies.

A critical epidemiological feature of FMDV is its propensity to establish persistent infection in exceeding 50% of infected ruminants, irrespective of vaccination status or clinical manifestations [5–7]. The persistent infection, commonly defined as the carrier status, is characterized by viral persistence within the pharynx beyond 28 days after viral exposure [8]. Importantly, oropharyngeal fluid (OPF) from carriers retains infectious, representing a transmission risk to susceptible cattle or buffalo [9–11]. The existence of FMDV carriers fundamentally undermines control and eradication efforts for FMD while imposing substantial barriers to regaining disease‐free status and resuming international livestock trade.

Significant advances have been made toward clarifying the pathogenesis and characteristics of persistent FMDV infection. The anatomical sites of viral persistence have been localized to the bovine nasopharyngeal epithelium [6, 12–15] and associated lymphoid tissues [16]. Several studies indicate that FMDV persistence correlates with suppressed antiviral innate immune responses [15] and impaired pro‐apoptotic signaling within the nasopharynx [17, 18]. Given that most FMDV proteins antagonize host innate immunity to facilitate viral replication and survival [19], it remains unclear whether the attenuation of local antiviral defenses is a prerequisite for, or a consequence of, persistent infection. This complexity is highlighted by observations showing downregulated antiviral type I interferon (IFN‐I) genes during acute FMDV infection in the nasopharynx [20]. Consequently, comprehensive longitudinal analyses of systemic immune responses may provide critical insights into determinants of FMDV persistence and aid in developing diagnostic tools to differentiate carriers from noncarriers.

FMDV infection or vaccination typically elicits robust humoral responses. Serological investigations revealed comparable antigen‐specific and neutralizing antibody titers between carriers and noncarriers [7, 21, 22], whereas notable differences in secretory IgA (sIgA) responses were observed in oral and nasal secretions during persistent infection [15, 23, 24]. In addition, CD4^+^ and CD8^+^ T cells perform diverse functions, including lysing virus‐infected cells, producing cytokines to coordinate immune responses, and aiding B‐cell activation for antibody production [25]. In vaccinated cattle, neutralizing antibody induction and immunoglobulin class switching rely on CD4^+^ T‐cell activity [26]. The comparable neutralizing antibody levels between carriers and noncarriers suggest that Th2‐mediated humoral responses are not the primary determinants of carrier status. Notably, the enhanced Th1 and cytotoxic CD8^+^ T‐cell responses have been associated with early protection against FMDV [27]. However, the contributions of the Th1‐mediated and cytotoxic T lymphocyte (CTL) responses to FMDV persistence remain elusive. Transcriptome analyses indicated that FMDV persistence correlated with impaired cell‐mediated immunity in the nasopharynx, with noncarriers exhibiting greater T‐cell infiltration than carriers [17, 18]. While previous investigations revealed no difference in circulating CD4^+^ and CD8^+^ T‐cell dynamics between carriers and noncarriers [7], the phenotypic profiles of distinct T‐cell subpopulations associated with FMDV infection or persistence remain largely uncharacterized.

This study aims to clarify host immune factors that differentiate vaccinated carriers from noncarriers during the early and persistent phases. We longitudinally assessed serological and mucosal antibody responses, along with peripheral lymphocyte subpopulations, from the initial FMDV challenge to the persistent/recovered phase. Carriers exhibited significantly lower frequencies of IFN‐*γ*‐producing CD4^+^ and CD8^+^ T subsets during early infection and diminished peripheral B‐cell frequencies alongside elevated OPF sIgA levels during persistent infection. These immunological signatures provide novel and practical bases for distinguishing persistently infected carriers from animals that have cleared FMDV.

## 2. Methods and Materials

### 2.1. Animal Experiment Design and Sample Collection

Fifteen 1‐year‐old bulls of a local breed from Gansu Province were prescreened and confirmed seronegative for FMDV prior to enrollment. All animals were vaccinated with a commercialized inactivated FMDV/O/BY/2010 (GenBank Accession No. JN998085.1) vaccine. At 21 days post‐vaccination (dpv), cattle were challenged intradermolingually with 10^4^ infectious doses of virulent FMDV/O/BY/2010. Clinical examination was performed every 2 days until 10 days post‐challenge (dpc) to monitor typical FMD vesicular lesions on the nasal epithelium, oral cavity, and feet. OPF samples were collected with probang cups at 7, 14, 21, 28, and 35 dpc. Nasal, oral, and anal swabs were immediately placed in tubes with 1 mL of DMEM containing 25 mM HEPES at 3, 7, 14, 21, 28, and 35 dpc. Blood samples were collected at −1, 3, 7, 14, 21, 28, and 35 dpc using disposable veterinary collection devices for serum isolation and EDTA‐anticoagulated tubes for peripheral blood mononuclear cell (PBMC) isolation. All samples were processed promptly by centrifugation to harvest serum and mucosal secretions. Probang samples were diluted 1 : 1 with DMEM (25 mM HEPES), homogenized and centrifuged to obtain supernatants. Aliquots from all sample types were frozen at −70°C for subsequent processing.

All animal experiments involving live FMDV in the present study were carried out in the animal biosafety level 3 (ABSL‐3) facility of Lanzhou Veterinary Research Institute, Chinese Academy of Agricultural Sciences. Experimental protocols were reviewed and approved by the Gansu Ethical Review Committee and complied with the standards and guidelines of the Gansu Animal Experiments Inspectorate (License No. SYXK‐GAN‐2018‐0005).

### 2.2. FMDV RNA Isolation and Detection

Total RNA was isolated from plasma, swab and OPF specimens (MiniBEST Viral RNA/DNA Extraction Kit, TaKaRa) and reverse transcribed into cDNA (PrimeScript RT Master Mix, TaKaRa) according to the supplier’s protocol. Viral load was quantified by real‐time PCR targeting the *FMDV 3D* gene [28]. Cycle threshold (*C*_T_) values were transformed into RNA copy numbers per *μ*L using a calibration curve generated from 10‐fold sequential dilutions of a recombinant plasmid pET28a‐FMDV/3D [27]. Viral load was expressed as Log_10_ gene copies/*μ*L.

### 2.3. Bovine PBMCs Isolation

PBMCs were separated from EDTA‐anticoagulated whole blood by density gradient centrifugation using Ficoll Plus 1.083 (Solarbio). Briefly, blood samples were diluted 1:1 with sterile 1× PBS (pH 7.4) and gently overlaid onto an equivalent volume of Ficoll Plus 1.083 in centrifuge tubes, ensuring distinct phase separation. Following centrifugation at room temperature at 800 × *g* for 30 min, mononuclear cells were collected from the plasma‐ficoll interface and transferred to fresh 15 mL tubes. The harvested cells were washed twice with sterile 1× PBS (pH 7.4) containing 2% fetal bovine serum (FBS, Gibco). Cell concentration and viability were determined before downstream analyses.

### 2.4. Virus Amplification and Purification

FMDV virions were purified as previously outlined [27] with minor modifications. Specifically, BHK‐21 cells were infected with FMDV/O/BY/2010 (MOI = 1) and harvested upon complete cytopathic effect (CPE; ~10 h postinfection). Infected cells underwent three freeze–thaw cycles to release virions, and lysates were clarified by centrifugation at 4°C with 10,000 × *g* for 30 min. The supernatant was incubated with 0.1%up][?tjl=1000][?down]? (v/v) binary ethylenimine (Sigma) at 30°C for 28 h under gentle agitation. Viral preparations were concentrated to ~20 mL by ultrafiltration through a Vivaflow 200 cassette with 100 kDa MWCO (Sartorius, Germany) and buffer‐exchanged into TNE buffer (0.05 M Tris‐0.1 M NaCl‐0.004 M EDTA, pH 8.0). The concentrated viral preparation was layered onto a continuous 10%–50% sucrose density gradient in TNE buffer and ultracentrifuged for 3 h with 250,000 × *g* at 4°C. Fractions were collected sequentially from top to bottom in 500 *μ*L aliquots, and those with peak absorbance at 260 nm were identified using NanoDrop (Thermo Fisher Scientific) (Supporting Information Figure S1A) and pooled. Viral particle integrity and morphology were confirmed by transmission electron microscopy (H‐7100FA, Hitachi) operated at 100 kV following negative staining with 2% phosphotungstic acid (pH 7.4) on 300 mesh copper grids (Polysciences, USA) (Supporting Information Figure S1B).

### 2.5. Flow Cytometry

Fluorochrome‐conjugated anti‐bovine antibodies and corresponding secondary antibodies employed in the study are detailed in Table 1. For T‐ and B‐cell surface phenotyping, PBMCs were blocked with 5% normal bovine serum (Jackson ImmunoResearch) for 20 min at 4°C to reduce nonspecific FC receptors binding [29]. Cells were stained first with viability dyes to exclude nonviable cells, then incubated with antibody cocktails at 4°C for 30 min, and washed with FACS buffer by centrifugation (10 min, 4°C, 300 × *g*).

**Table 1:** Antibodies used in spectral flow cytometry experiments.

Marker	Conjugated	Labeling strategy	Target species	Clone	Host	Isotype	Catalog no.	Manufacturer
CD21	AF647	Direct conjugate	Bovine	CC51	Mouse	IgG2b	MCA5953A647	Abd
CD3	BV605	Secondary antibody	Bovine	MM1A	Mouse	IgG1	MCA6080	Abd
CD4	AF700	Direct conjugate	Bovine	CC8	Mouse	IgG2a	MCA1653A700	Abd
CD8	AF647	Direct conjugate	Bovine	CC63	Mouse	IgG2a	MCA837A647	Abd
*γδ*TCR	PerCP‐Cy5.5	Direct conjugate	Bovine	GB21A	Mouse	—	WSC0578B‐100	Kingfisher
CD44	FITC	Direct conjugate	Bovine	IL‐A118	Mouse	IgG1	MCA2433F	Abd
CD62L	PE	Direct conjugate	Bovine	CC32	Mouse	IgG1	MCA1649G	Abd
IFN‐*γ*	RPE	Direct conjugate	Bovine	CC302	Human	IgG1	MCA1783PE	Abd
Fixable viability dye	eFluor 780	Direct conjugate	All species	—	—	—	65‐0865	eBioscience

For intracellular cytokine staining, PBMCs were adjusted to 1 × 10^6^ cells per 100 *μ*L and stimulated with 10 *μ* g/mL sucrose gradient‐purified 146S FMDV/O/BY/2010 particles at 37°C for 8 h. Brefeldin A (Biolegend) at 1 *μ*g/mL final concentration was added during the last 6 h to block protein secretion. Surface staining was performed as described above, followed by fixation and permeabilization using a BD Fixation/Permeabilization Solution Kit according to the supplier’s instructions. Intracellular IFN‐*γ* was detected utilizing specific monoclonal antibodies. Data were acquired on a CytExpert flow cytometer (Beckman) and analyzed in FlowJo software (TreeStar). Doublets were excluded by FSC‐A versus FSC‐H gating. Analysis was restricted to live, single events, with 50,000 events collected per sample for statistical analysis.

### 2.6. Serology Detection

Total FMDV‐specific IgG levels were quantified using a commercial lpELISA Kit (LSBIO, Lanzhou, China) following the manufacturer’s protocol.

Neutralizing antibody titers were measured using a conventional microplate‐based virus‐neutralization assay. Specifically, sera were incubated at 56°C for 30 min to abolish complement activity, two‐fold serially diluted in DMEM (Sigma), plated into 96‐well plates (50 *μ*L per well), and mixed with identical volumes of 100 TCID_50_ of FMDV/O/BY/2010. Following virus‐serum incubation at 37°C for 1 h, BHK‐21 cell suspensions (2.5 × 10^4^ cells in 50 *μ*L per well) were plated and cultured for 72 h. CPE was examined microscopically, and neutralizing antibody titers were estimated using the Reed and Muench method [30].

The levels of sIgA in OPF was detected by indirect ELISA. Briefly, 96‐well microplates were coated with 2 *μ*g/mL sucrose gradient purified 146S FMDV/O/BY/2010 particles overnight at 4°C, then blocked with 1% (w/v) BSA in PBS for 1 h at 37°C. OPF samples diluted 1:4 in PBST (0.05% v/v Tween‐20 in PBS) were loaded and incubated at 37°C for 1 h, followed by incubation with anti‐bovine IgA HRP‐conjugated MAbs (AbD‐Serotec) diluted 1 : 2,000 in PBST for 1 h at 37°C. Following three rinses with PBST, 100 *μ*L of TMB substrate was added per well. The reaction was developed in darkness for 10–30 min at ambient temperature and terminated with 50 *μ*L of 0.2 M H_2_SO_4_. The absorbance at 450 nm was measured using a spectrophotometer (Biotek, Vermont, USA).

### 2.7. Correlation Analysis and Evaluation of Candidate Diagnostic Biomarkers

The correlation between OPF sIgA levels and peripheral B‐cell frequencies was evaluated using linear regression. Diagnostic efficacy was estimated by the receiver operating characteristic (ROC) curve analysis. The area under the curve (AUC) with 95% confidence interval (CI), as well as diagnostic specificity and sensitivity were estimated using MedCalc software (Version 23.1.2). AUCs > 0.9 were considered indicative of excellent diagnostic potential.

### 2.8. Statistical Analysis

Data processing and figure generation were performed in GraphPad Prism software (Version 9.0). Results were displayed as mean ± SEM. Statistical significance was determined by two‐tailed paired student’s *t*‐tests, with *p* < 0.05 considered significant.

## 3. Results

### 3.1. Infection Dynamics of Vaccinated Carriers and Noncarriers

To investigate the occurrence and characteristics of persistent FMDV infection in vaccinated cattle, challenge experiments were conducted on 15 cattle at 21 dpv. Throughout the entire 35‐day sampling period following viral challenge, *FMDV 3D* RNA loads in OPF, plasma, nasal swabs, oral swabs, and anal swabs from each animal was continuously monitored to assess infection dynamics and viral persistence. None of the vaccinated cattle developed clinical signs after challenge, and viral RNA was rarely detectable in plasma. Continuous monitoring of OPF revealed that eight cattle (53.3%) remained positive for *FMDV 3D* RNA throughout the entire sampling period and were defined as carriers. In contrast, seven cattle (46.7%) showed undetectable viral RNA in OPF by 28 dpc and were designated noncarriers. Furthermore, *FMDV 3D* RNA was undetectable in nasal, oral, and anal swabs in both groups by 28 dpc (Figure 1A,B). These viral shedding patterns observed here are consistent with previous reports [6, 15], confirming the successful establishment of a persistent FMDV infection model in vaccinated cattle.

Figure 1FMDV infection dynamics in vaccinated cattle. FMDV RNA loads in FMDV carriers (*n* = 8) (A) and noncarriers (*n* = 7) (B). The FMDV RNA loads (mean log_10_ copies per *u*L ± SD) in plasma from 3 to 28 dpc, in OPF samples from 7 to 35 dpc, as well as in oral, nasal, and anal swabs from 3 to 35 dpc were measured. CR, carriers; dpc, days post‐challenge; NCR, noncarriers.

**Figure:**
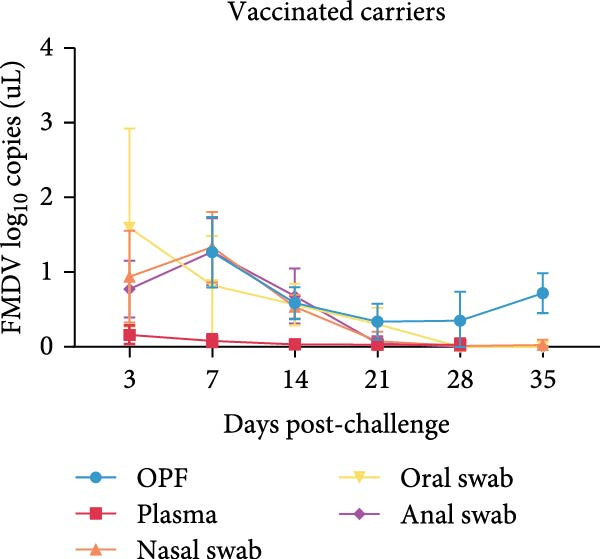
(A)

**Figure:**
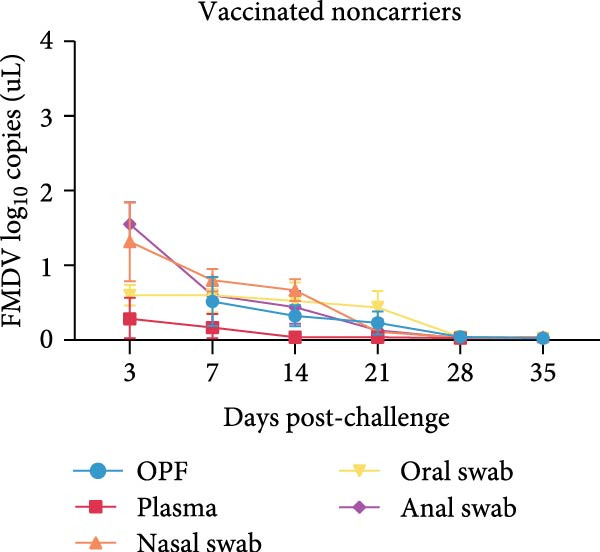
(B)

### 3.2. Reduced Prevalence of IFN‐*γ*‐Expressing CD4^+^ and CD8^+^ T Subsets in Carriers During the Early Infection Phase

Given the association between chronic infections and impaired T‐cell activation and differentiation [31], we assessed the phenotypic profiles of T‐cell subpopulations in carriers relative to noncarriers. The differential expression patterns of adhesion molecules CD44 (H‐CAM) and CD62L (L‐selectin) function as phenotypic markers for distinguishing naive (CD62L^high^CD44^low^), central memory (CD62L^high^CD44^high^), and effector/memory (CD62L^low^CD44^high^) T‐cell subsets [32, 33]. Using the gating strategies illustrated in Figure 2A, flow‑cytometric analyses indicated no significant differences between carriers and noncarriers in the dynamics of *α*β T‐cell populations (CD4^+^ and CD8^+^ gated in CD3^+^ *γδ*TCR^−^) and *γδ* T‐cell population (CD3^+^ *γδ*TCR^+^) (Figure 2B), nor in their respective naive, central memory, and effector/memory subpopulations (Figure 3A–C), suggesting comparable activation and differentiation kinetics in both groups.

Figure 2T‐cell populations diverge between carriers and noncarriers. (A) The basic gating strategies to define bovine T‐cell subsets. The lymphocytes are chosen with FSC versus SSC and then single cells are gated using FSC‐A and FSC‐H. Dead cells were excluded by outputting eFlour 780. By outputting CD3 versus *γδ*TCR, CD3^+^ *γδ*TCR^−^ and CD3^+^ *γδ*TCR^+^ populations are identified, respectively. By outputting CD4 versus CD8 in the CD3^+^ *γδ*TCR^−^ population, CD3^+^ *γδ*TCR^−^CD4^+^CD8^−^ and CD3^+^ *γδ*TCR^−^CD4^−^CD8^+^ T cells are identified, respectively. The CD3^+^ *γδ*TCR^−^CD4^+^CD8^−^, CD3^+^ *γδ*TCR^−^CD8^+^CD4^−^, and CD3^+^ *γδ*TCR^+^ T cells contains CD62L^high^CD44^low^, CD62L^high^CD44^high^, and CD62L^low^CD44^high^ subpopulations. (B) The proportions of CD3^+^ *γδ*TCR^+^ in lymphocytes, as well as CD4^+^ and CD8^+^ subsets gated in CD3^+^ *γδ*TCR^−^ populations. CR, carriers; FSC, forward scatter; NCR, noncarriers; SSC, side scatter.

**Figure:**
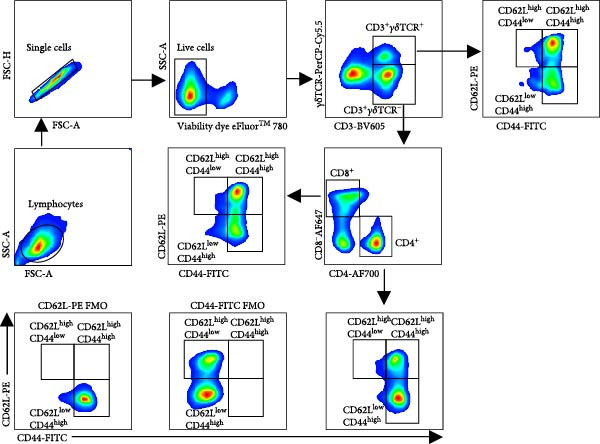
(A)

**Figure:**
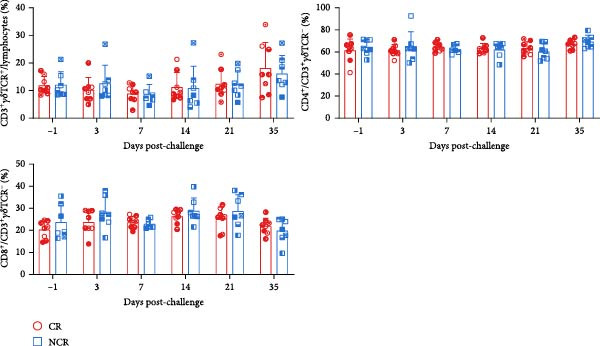
(B)

Figure 3T‐cell subpopulations diverge between carriers and noncarriers. The proportions of CD62L^high^CD44^low^, CD62L^high^CD44^high^, and CD62L^low^CD44^high^ subpopulations in CD3^+^ *γδ*TCR^−^CD4^+^CD8^−^ (A), CD3^+^ *γδ*TCR^−^CD4^−^CD8^+^ (B), and CD3^+^ *γδ*TCR^+^ (C) populations. CR, carriers; NCR, noncarriers.

**Figure:**
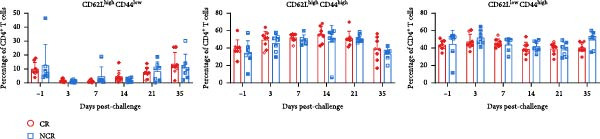
(A)

**Figure:**
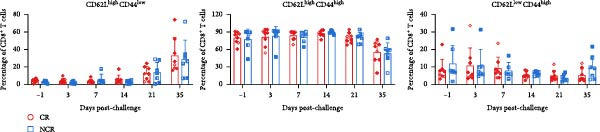
(B)

**Figure:**
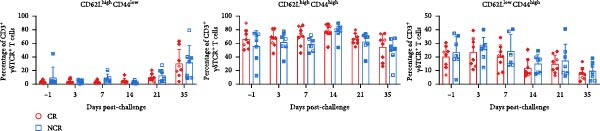
(C)

Antigen‐activated T cells promote further differentiation, proliferation, and cytolytic activity of immune cells through cytokine secretion, with IFN‐*γ* production being a key indicator of Th1‐type and CTL responses in cattle or swine following FMD vaccination. Accordingly, the proportions of IFN‐*γ*‐positive CD4^+^ and CD8^+^ T cells were assessed in carriers relative to noncarriers, as detailed in the gating scheme in Figure 4A. Both IFN‐*γ*‐secreting CD4^+^ and CD8^+^ frequencies elevated post‐challenge, peaked at 14 dpc, and then declined in both groups. Notably, carriers displayed markedly lower frequencies of IFN‐*γ*‐expressing CD4^+^ at 14 dpc (*p* = 0.033) and CD8^+^ T cells at 7 dpc (*p* = 0.015) than noncarriers (Figure 4B,C). These findings indicate attenuated Th1/CTL responses in carriers, which may hinder viral clearance and predispose to persistence.

Figure 4IFN‐*γ*‐producing T subsets diverge between carriers and noncarriers. (A) The basic gating strategies to define bovine IFN‐*γ*‐producing T subsets. The lymphocytes are chosen with FSC versus SSC, and then single cells are gated using FSC‐A and FSC‐H. Dead cells were excluded by outputting eFlour 780. CD3^+^ T cells are identified by expressing CD3. By outputting CD4 versus CD8 in the CD3^+^ T cells, CD4^+^CD8^−^ and CD8^+^CD4^−^ T cells are identified, respectively. IFN‐*γ*‐producing cells are identified by outputting IFN‐*γ* in CD4^+^CD8^−^ (B) and CD8^+^CD4^−^ (C) T cells.  ^∗^ indicates *p* < 0.05. CR, carriers; FSC, forward scatter; NCR, noncarriers; SSC, side scatter.

**Figure:**
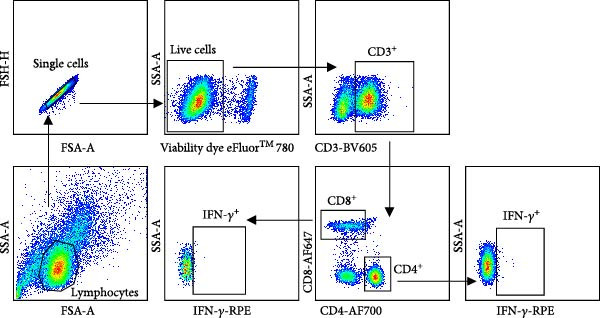
(A)

**Figure:**
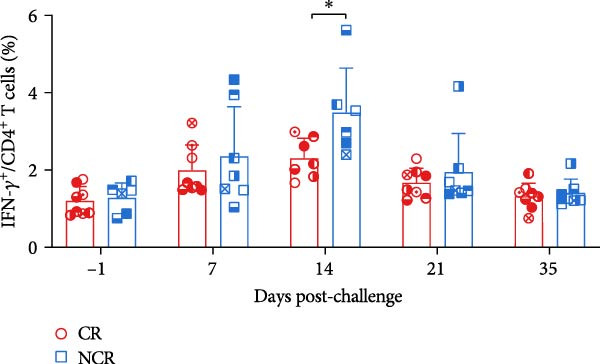
(B)

**Figure:**
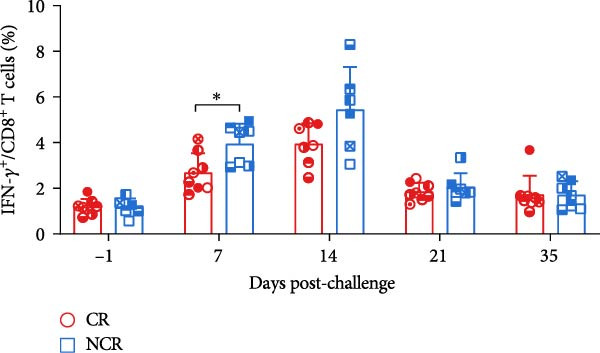
(C)

### 3.3. Decreased Peripheral B‐Cell Frequencies in Carriers During the Persistent Infection Phase

B lymphocytes are key mediators of antibody production, with CD21 expression restricted to B cells in cattle [34]. Using the gating approach illustrated in Figure 5A, we found that peripheral B‐cell frequencies declined transiently post‐challenge, followed by recovery from 14 dpc in noncarriers. In contrast, B‑cell frequencies in carriers remained unchanged or failed to recover from 14 dpc. This divergence resulted in significantly lower peripheral B cell frequencies in carriers compared to noncarriers at 21 dpc (*p* = 0.022) and 35 dpc (*p* = 0.003) (Figure 5B).

Figure 5B‐cell population diverge between carriers and noncarriers. (A) The basic gating strategy for bovine B cells. The lymphocytes are chosen with FSC versus SSC, and then single cells are gated using FSC‐A and FSC‐H. Dead cells were excluded by outputting eFlour 780. By outputting AF647, CD21^+^ B cells are identified. (B) The proportion of CD21^+^ B cells in lymphocytes.  ^∗^ indicates *p* < 0.05.  ^∗∗^ indicates *p* < 0.01. CR, carriers; FSC, forward scatter; NCR, noncarriers; SSC, side scatter.

**Figure:**
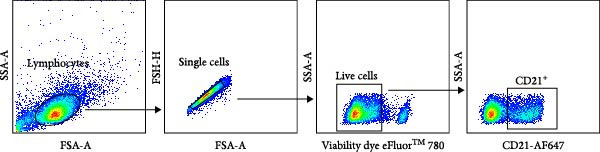
(A)

**Figure:**
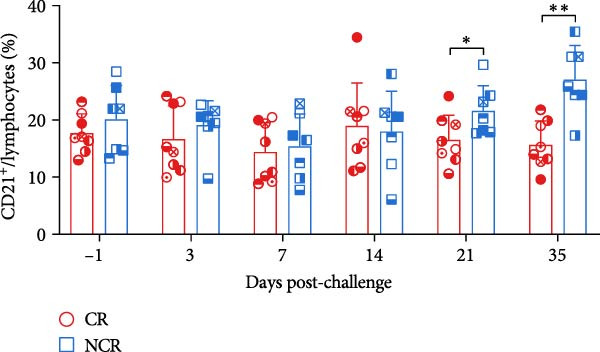
(B)

### 3.4. Elevated OPF sIgA Levels in Carriers During the Persistent Infection Phase

We next compared longitudinal serum antibody profiles between carriers and noncarriers. The results revealed no significant differences in either antigen‐specific or neutralizing antibody levels between the two groups at any post‐challenge time point (Figure 6A,B), consistent with previous reports [7, 21, 22]. These findings confirm that systemic serological responses cannot discriminate between carrier and noncarrier states.

Figure 6The dynamics of serological and mucosal antibody titers of carriers and noncarriers after challenge. The dynamics of antigen‐specific antibody (A), neutralizing antibody (B) in serum, and sIgA (C) in OPF.  ^∗^ indicates *p* < 0.05. CR, carriers; NCR, noncarriers.

**Figure:**
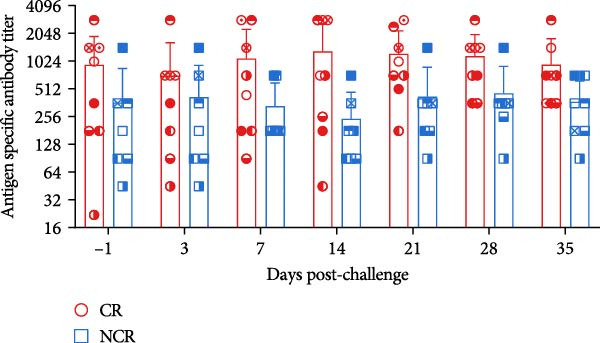
(A)

**Figure:**
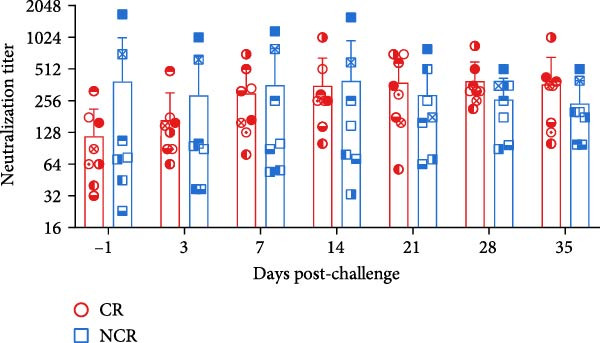
(B)

**Figure:**
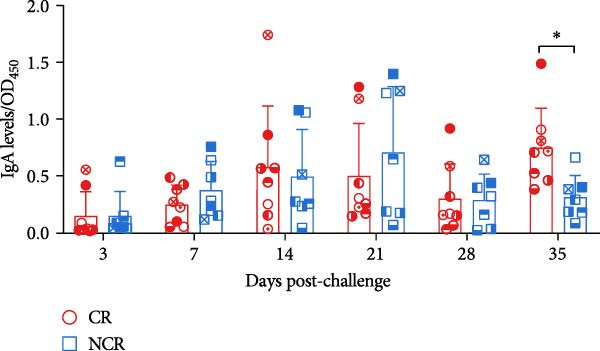
(C)

Given that FMDV transmission occurs primarily through respiratory routes in cattle, we further evaluated the sIgA kinetics in OPF. Distinct patterns emerged between the two groups. In noncarriers, sIgA levels peaked at 21 dpc, then declined and stabilized. However, in carriers, sIgA levels remained elevated beyond 28 dpc, resulting in remarkably higher sIgA levels at 35 dpc (*p* = 0.017) (Figure 6C). This kinetics is consistent with the pattern of viral shedding in OPF, suggesting that elevated sIgA levels in OPF is associated with viral replication. Notably, this sustained mucosal response in carriers paradoxically coincided with reduced peripheral B‐cell frequencies, suggesting possible immune compartmentalization or recruitment of B cells to secondary lymphoid structures in the upper respiratory tract in response to ongoing viral replication.

### 3.5. Diagnostic Potential of the Combination of Peripheral B‐Cell Frequencies and OPF sIgA Levels for FMDV Carrier Identification

To explore the relationship between persistent mucosal antibody elevation and peripheral B‐cell depletion, we conducted a correlation analysis. The results demonstrated a significant negative correlation between OPF sIgA levels and circulating B‑cell frequencies (Figure 7A), suggesting a potential diagnostic synergy for identifying FMDV persistence. Subsequently, ROC analyses were performed to evaluate the diagnostic performance of each marker. Both OPF sIgA levels and peripheral B‐cell frequencies demonstrated high diagnostic accuracy, with AUC values of 0.920 (95% CI: 0.662–0.997) and 0.964 (95% CI: 0.725–1.000), respectively (Figure 7B).

Figure 7Evaluation of signatures differentiating carriers from noncarriers. (A) The correlation (with 95% confidence intervals) between the proportion of CD21^+^ B cells in peripheral blood and sIgA levels in OPF. The two‐sided *p*‐values were determined by Pearson’s correlation test. (B) The area under the curve (AUC) represents diagnostic performance. ROC curves for the proportion of CD21^+^ B cells in peripheral blood (green), sIgA levels in OPF (orange), and the combination of these two markers (blue). CR, carriers; NCR, noncarriers.

**Figure:**
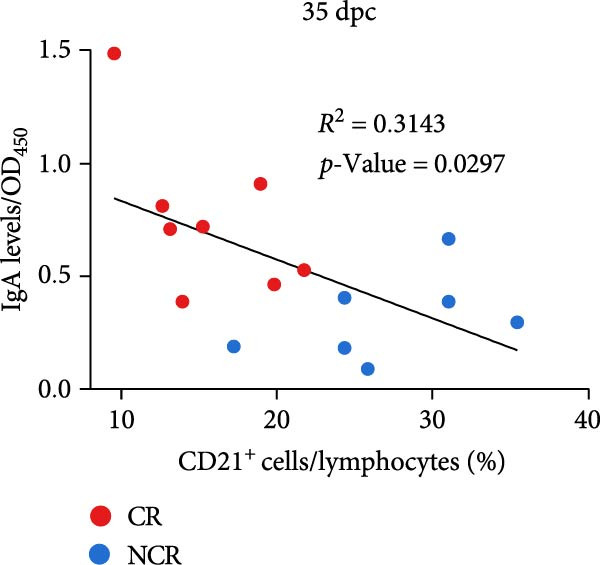
(A)

**Figure:**
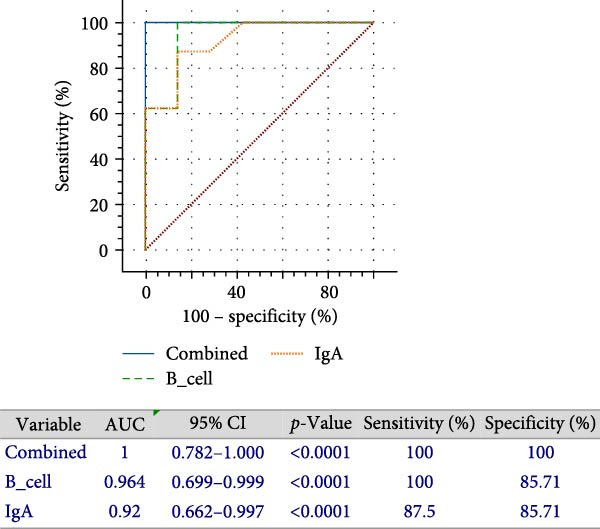
(B)

To assess the combined diagnostic utility, a binary logistic regression model incorporating both markers achieved an AUC of 1.000 (95% CI: 0.782–1.000), with 100% specificity and sensitivity (Figure 7B). These findings underscore that simultaneous measurement of peripheral B‐cell frequencies and OPF sIgA levels enables precise differentiation of persistently infected cattle, offering substantial practical utility for FMD management and control in livestock. Given the unresolved dynamics of total circulating B‐cell kinetics in naïve carriers versus noncarriers, further investigations are warranted to evaluate their pivotal role in persistent FMDV infection and their potential as supplementary diagnostic indicators.

## 4. Discussion

This study provides a comprehensive characterization of systemic and mucosal immune responses in vaccinated cattle following FMDV challenge, aiming to identify immune correlates associated with viral persistence versus clearance. Extending previous work [7], we conducted comprehensive comparative analyses of B‐ and T‐lymphocyte subsets, serological and mucosal antibody kinetics, addressing prior gaps in characterization of B and T‐subset phenotypes in vaccinated carriers and noncarriers. Distinct immunological signatures were observed between carriers and noncarriers. Carriers exhibited significantly lower proportions of IFN‐*γ*‐expressing CD4^+^ and CD8^+^ T cells during early infection, accompanied by reduced peripheral B‐cell frequencies and elevated OPF sIgA levels during persistent infection.

Consistent with previous reports [7, 21, 22], systematic antigen‐specific IgG and neutralizing antibody titers were comparable between carriers and noncarriers, underscoring their limited utility as discriminative diagnostic markers. In contrast, mucosal sIgA has been consistently recognized as a reliable indicator of FMDV carrier status [23, 24, 35, 36]. Our findings corroborate this diagnostic utility, demonstrating sustained OPF sIgA levels in vaccinated carriers during persistent infection. Antibody‐mediated protection against FMDV depends primarily on plasma and memory B cells. Earlier studies indicated that circulating FMDV‐specific IgG‐secreting cells peak within 7–14 dpc in both naïve and vaccinated cattle, and then decline to minimal or undetectable levels [7, 37]. This kinetics does not mirror changes in circulating antibody levels or mucosal sIgA profiles, potentially reflecting the swift activation of FMDV‐specific plasma cells within secondary lymphoid tissues surrounding the upper respiratory tract following viral challenge [38]. However, the overall dynamics of total circulating B‐cell populations after FMDV exposure remain inadequately characterized and merit further investigation.

In our investigation, carriers exhibited significantly lower peripheral CD21^+^ B‐cell frequencies compared with noncarriers during the persistent phase. This reduction, coinciding with elevated sIgA levels and viral loads in OPF, suggests potential recruitment of B cells to infection sites or associated secondary lymphoid organs, likely reflecting localized nasopharyngeal immune activation in response to ongoing viral replication. Notably, combining peripheral B cell frequencies with OPF sIgA levels significantly enhanced diagnostic specificity and sensitivity relative to either marker alone, enabling more accurate carrier identification. These observations underscore the potential of integrated systemic and mucosal immune profiling for precise identification of persistently infected cattle, offering tangible benefits for FMD surveillance and control. Future work should prioritize longitudinal analyses of peripheral B‑cell dynamics in larger animal cohorts, as well as in unvaccinated carriers and noncarriers to further elucidate their critical role in the pathogenesis of FMDV persistence and evaluate their potential as supplementary diagnostic biomarkers.

Accumulative evidence highlights the pivotal role of T lymphocyte–mediated immunity—especially CD8^+^ subsets—in early viral control and cross‐protective responses in cattle [27, 39–41]. Previous reports have shown that non‑carriers possess higher T‐cell abundance in nasopharyngeal tissues than FMDV carriers [15], and stronger cellular responses correlate positively with the absence of viral shedding in OPF samples [42]. In pigs, FMDV infection induces pronounced lymphopenia and immunosuppression, characterized by substantial reductions in circulating T lymphocytes [43, 44]. In cattle, hematological responses appear more variable. While several investigations document significant lymphopenia during acute infection in naïve cattle [7, 45, 46], others reveal no appreciable alterations in total circulating leukocyte counts or T‐lymphocyte subset distributions in either naïve [47, 48] or vaccinated cattle [7]. In our study, frequencies of peripheral CD4^+^ and CD8^+^ subsets in vaccinated cattle remained largely unchanged following viral challenge, with no significant differences in their repective activation or differentiation signatures between carriers and noncarriers throughout the study period. Interestingly, carriers exhibited significantly lower frequencies of IFN‐*γ*‐expressing CD4^+^ and CD8^+^ T lymphocytes during early infection than noncarriers. Th1 cells producing IFN‐*γ* are associated with higher titers or affinity of neutralizing antibody and better protection against FMDV [27]. Furthermore, the differentiation of CD8^+^ effector T cells producing IFN‐*γ* critically depends on IFN‐*γ*‐positive CD4^+^ T cells, as demonstrated in mycobacterial infection models [49]. The observed correlation between IFN‐*γ*‐expressing CD4^+^ and CD8^+^ subsets during FMDV infection [27] supports a cooperative role in early viral clearance. Together, these findings indicate compromised Th1/CTL responses in carriers, which facilitates viral persistence. Vaccines optimized to enhance Th1‐biased immunity and effective CD8^+^ T‐cell responses potentially reduce the likelihood of persistent FMDV infection.

In cattle, *γδ* T lymphocytes are highly abundant, constituting approximately 60% of circulating T cells [50]. Although proposed to possess NK‐like or CTL‐like functions, only a minor fraction produces IFN‐*γ* and/or perforin upon activation by bacterial, viral, or nonspecific stimuli [51]. More recent studies indicate that bovine *γδ* T cells represent a major regulatory and suppressive T‐cell subset [52]. In this study, both the frequencies and activation/differentiation profiles of *γδ* T cells remained comparable between carriers and noncarriers throughout the study period. Functional assays are warranted to define its contribution to FMDV persistence in either naïve or vaccinated cattle. Transcriptomic analyses of nasopharyngeal tissues from FMDV carriers have revealed significant upregulation of multiple inhibitory molecules associated with T‐cell exhaustion, including LAG‐3, Tim‐3, BTLA, CTLA‐4, NFAT1, BATF, and eomesodermin [18]. These findings underscore the complexity of T‑cell responses during viral persistence and highlight the need for deeper investigation into subset‐specific dynamics. Advanced approaches, such as targeted multi‑omics profiling or single‐cell omics, hold considerable potential for elucidating molecular mechanisms underlying persistent infection, particularly in livestock species historically constrained by limited immunological research tools.

In addition, sex‐based differences in immune responses are known to influence susceptibility and clinical outcomes in infectious diseases [53]. Notably, sex has been identified as a significant risk factor for FMDV susceptibility [54–56], underscoring the importance of incorporating sex as a biological variable in future studies of persistent FMDV infection.

## 5. Conclusions

In summary, this study provides a comprehensive characterization of systemic and mucosal immune responses in vaccinated cattle throughout the entire post‑challenge period. Although CD4^+^, CD8^+^, and *γδ* T cells demonstrated comparable activation and differentiation kinetics in both groups, carriers showed pronounced reductions in IFN‐*γ*‐expressing CD4^+^ and CD8^+^ T‐cell frequencies during early infection. This diminished IFN‐*γ* responses likely impair effective virus clearance and facilitate persistence. During persistent infection, carriers displayed distinct immunological profiles characterized by decreased peripheral B‐cell frequencies alongside elevated OPF sIgA levels. The combination of these two parameters provided greater diagnostic accuracy for carrier identification than either parameter alone. Collectively, these findings advance the current understanding of the cellular and humoral features of FMDV persistence and provide a rational basis for the development of more precise diagnostic strategies. Ongoing work in our laboratory seeks to delineate the molecular dynamics of lymphocyte subsets and the immune modulators in FMDV persistence. Insights gained from these studies will inform the rational design of next‑generation vaccines and refined control strategies to mitigate viral persistence in livestock populations.

## Disclosure

All authors reviewed and approved the submitted manuscript.

## Conflicts of Interest

The authors declare no conflicts of interest.

## Author Contributions

Conceptualization: Huichen Guo, Shiqi Sun, and Zhihui Zhang. Supervision: Huichen Guo, Shiqi Sun, and Yijing Li. Project administration: Huichen Guo and Shiqi Sun. Sampling: Zhidong Teng, Suyu Mu, Shuanghui Yin, and Yaozhong Ding. Investigation: Zhihui Zhang, Zhidong Teng, Shuang Wang, Sumin Wei, Suyu Mu, Sumin Wei, Yun Zhang, Shuanghui Yin, and Hu Dong. Data analysis: Zhihui Zhang and Zhidong Teng. Visualization: Zhihui Zhang. Writing – original draft preparation: Zhihui Zhang. Writing review and editing: Huichen Guo, Shiqi Sun, and Zhihui Zhang.

## Funding

This work was supported by the National Natural Science Foundation of China (32473012, 32301127), National Key R&D Program of China (2021YFD1800303), Key R&D Program of Gansu province (25YFNA014), Key R&D Program of Ningxia province (2024BBF02017), Natural Science Foundation of Gansu Province (23JRRA551, 24JRRA012), and China Postdoctoral Science Foundation Funded Project (2024M763620).

## Supporting Information

Additional supporting information can be found online in the Supporting Information section.

## Supporting information


**Supporting Information** Figure S1. Purification and characterization of FMDV virions.

## Data Availability

The data that support the findings of this study are available from the corresponding author upon reasonable request.
