# Supplementary material for: Distinctive Immunological Signatures Define Foot‐and‐Mouth Disease Virus Persistence in Vaccinated Cattle
Source: Transbound Emerg Dis. 2025 Dec 28;2025:4010309. doi: 10.1155/tbed/4010309 (PMC12745507; doi:10.1155/tbed/4010309)
Supplement: Supplementary file 1 — Supporting Information Figure S1. Purification and characterization of FMDV virions. [file TBED-2025-4010309-s001.docx]

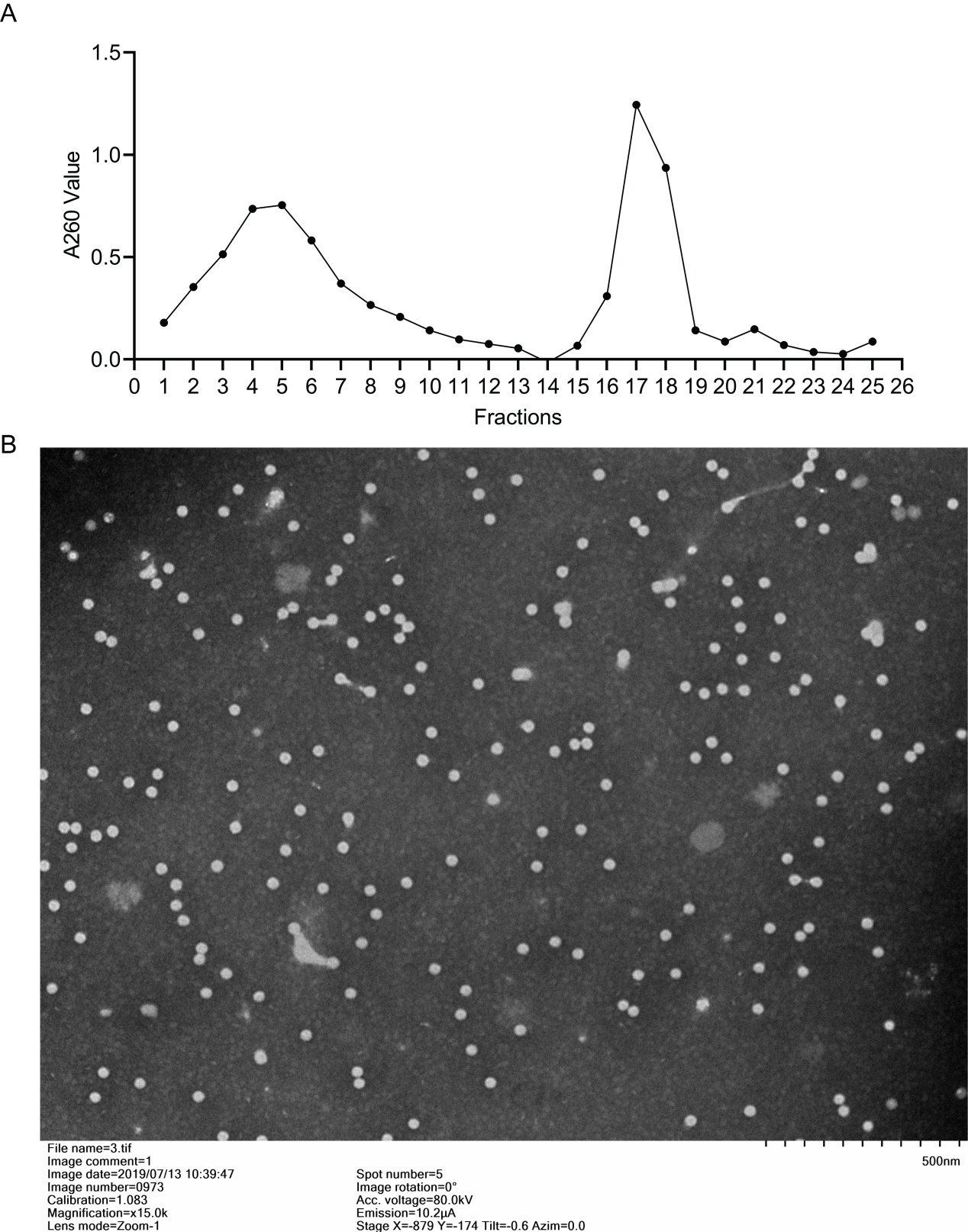


**Figure S1. Purification and characterization of FMDV virions.** (A) *OD_260_* peak values collected from a 10 to 50% sucrose density gradient demonstrates the presence of virions. (B) A negatively stained electron micrograph of the fractions with *OD_260_* peak values.
